# Supplementary figures and images for: High-throughput sequencing of EcoWI restriction fragments maps the genome-wide landscape of phosphorothioate modification at base resolution
Source: PLoS Genet. 2022 Sep 19;18(9):e1010389. doi: 10.1371/journal.pgen.1010389 (PMC9521924; doi:10.1371/journal.pgen.1010389)

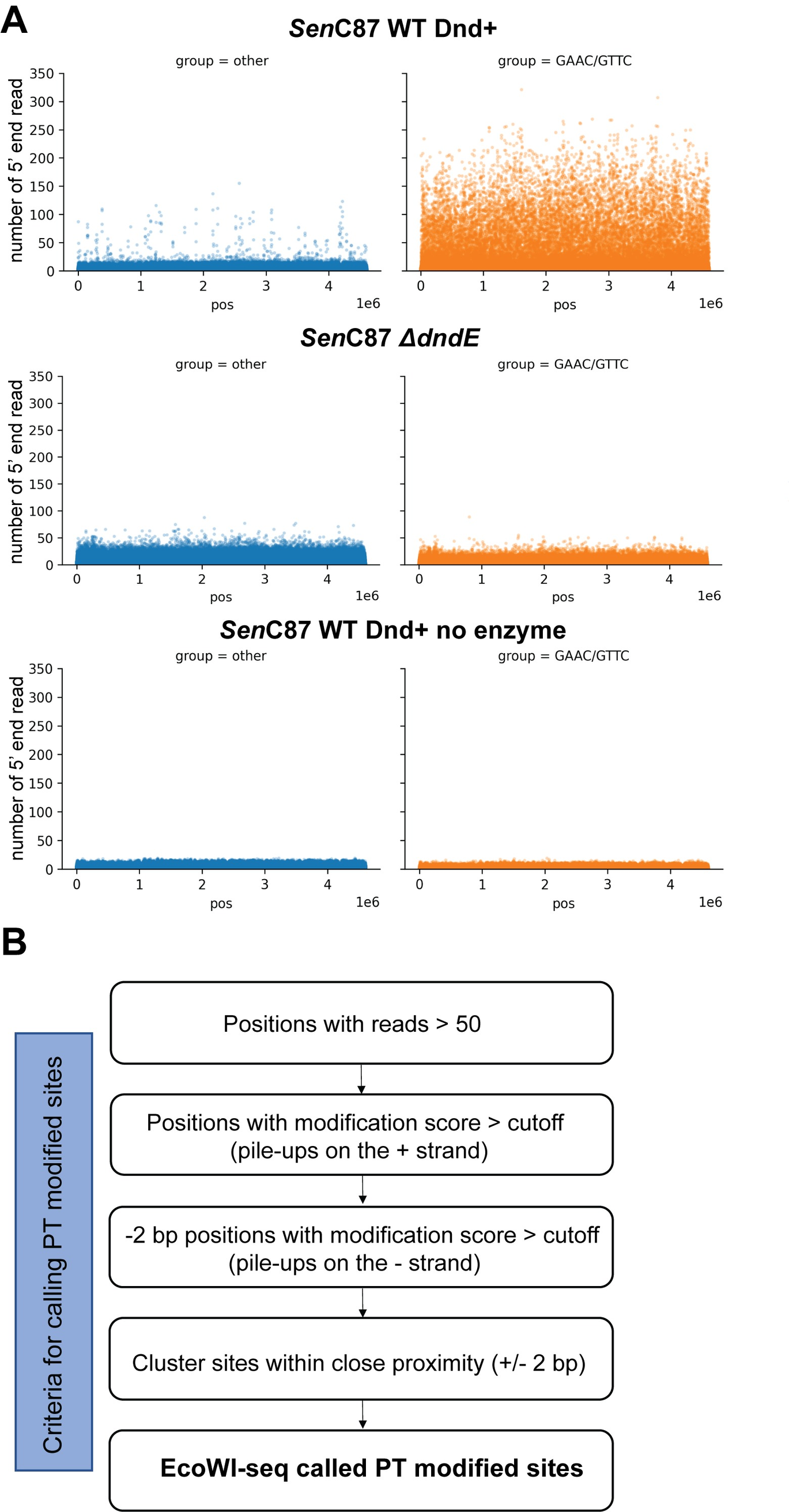

Supplement: S1 Fig — (A) Plots of the 5’ end reads coverage across all genomic positions. Top: EcoWI-seq on WT Dnd+ Sen 87 strain; middle: EcoWI-seq on ΔdndE mutant strain; bottom: Sequencing of WT Dnd+ SenC87 strain without EcoWI treatment. (B) Schema describing the analytical steps for PT-modified sites identification using EcoWI-seq. (TIF) [file pgen.1010389.s001.tif]

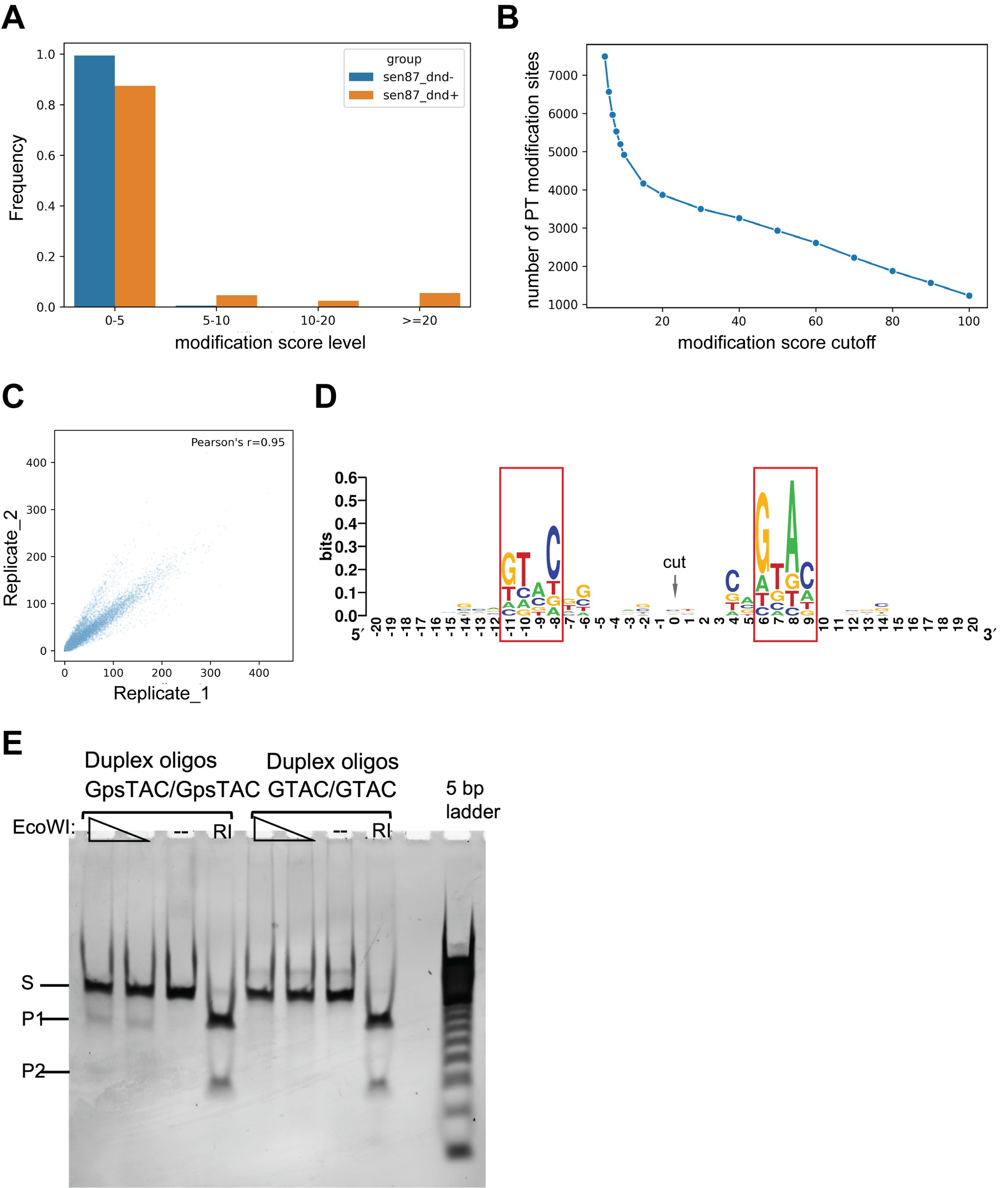

Supplement: S2 Fig — (A) Frequencies of modification level at all possible GAAC/GTTC sites for WT Dnd+ (orange) and ΔdndE mutant (blue) SenC87 strains. (B) Curve showing the number of PT modified sites identified at different modification score cutoff. The cutoff for calling modified sites in this experiment was determined by visual observation of the deviation from the tangent point. (value = 20). (C) Scatter plot showing correlation of modification scores in two independent replicates. Each dot represents a GAAC/GTTC site. (D) Motif logo of the flanking regions of non-canonical GAAC/GTTC sites. Positions are relative to the predicted cutting site (position 0). (E) EcoWI digestion of PT-modified duplex oligos containing GpsTAC or unmodified GTAC site. S, P1, and P2 refer to substrate (58mer), cleavage products 1 and 2, respectively. EcoWI digest condition, PAGE analysis, DNA fragment staining and imaging were described in the Materials and Method. EcoRI-HF (RI) was used as a positive control for restriction. (TIF) [file pgen.1010389.s002.tif]

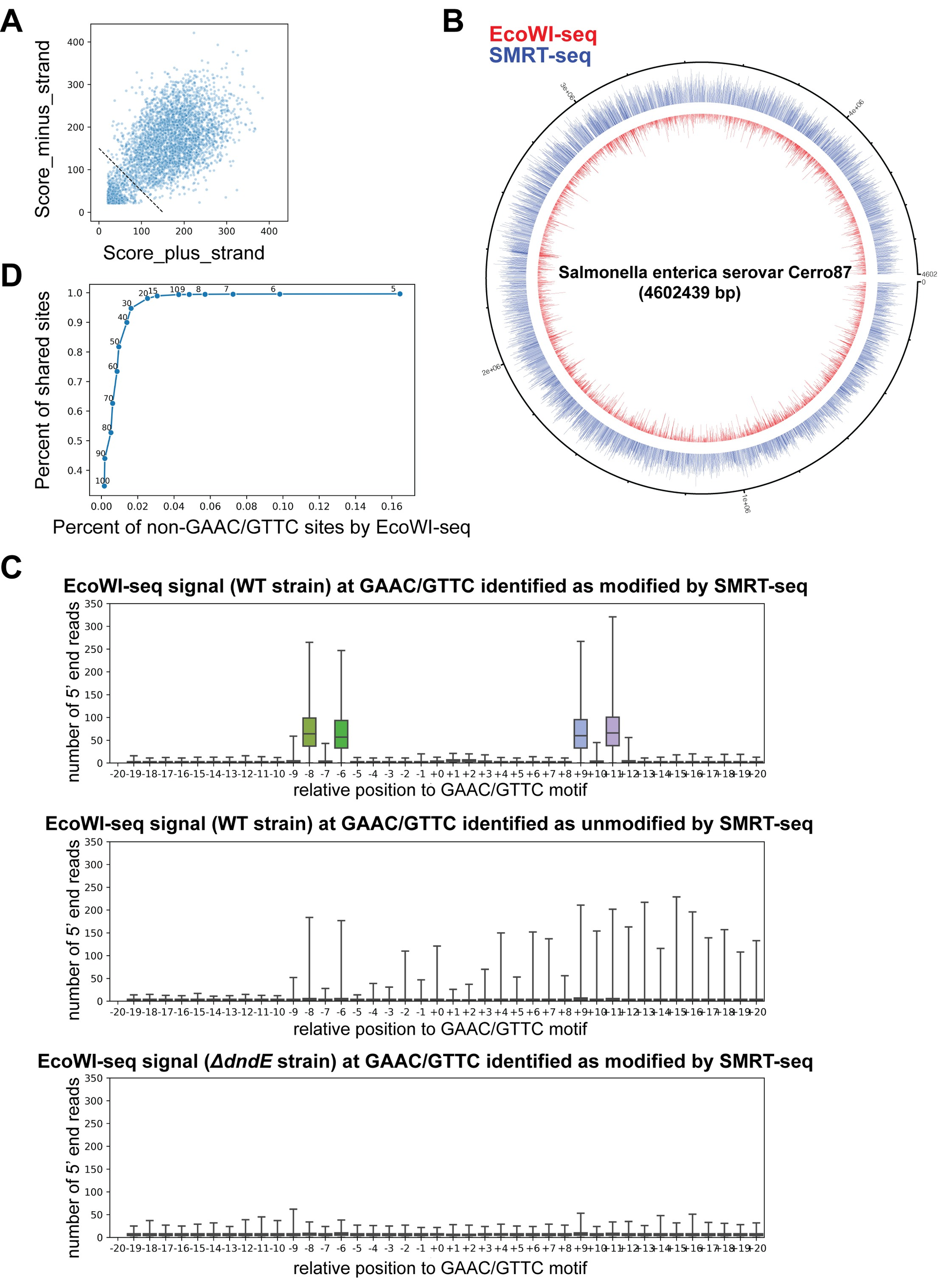

Supplement: S3 Fig — (A) Kinetic signals of GAAC/GTTC sites across the genome of the WT Dnd+ SenC87 strain using SMRT-sequencing. The dashed line displayed the threshold cutoff for calling modified sites (16). (B) Genome-wide circos plot showing distribution of PT modifications in WT Dnd+ SenC87 strain. The height of the bars represented the modification scores in EcoWI-seq (red) and kinetics signals in SMRT sequencing (blue). Only called modification sites were displayed. (C) Plots showing number of 5’ end reads in EcoWI-seq data across GAAC/GTTC motifs. Positions were relative to the GAAC/GTTC motif (+0 was set as the position of the G). Top: EcoWI-seq signal in the WT Dnd+ strain for the 3473 GAAC/GTTC sites called as modified by SMRT sequencing; middle: EcoWI-seq signal in the WT Dnd+ strain for 3500 randomly selected sites called as unmodified by SMRT sequencing; bottom: EcoWI-seq signal in the ΔdndE mutant strain for the 3473 GAAC/GTTC sites called as modified by SMRT sequencing using the WT Dnd+ strain. (D) Curve with Y axis represents the overlapping percent of modified sites called by EcoWI-seq and SMRT sequencing (percentage relative to number of modified sites in SMRT sequencing) and X axis represents the percent of identified non-canonical GAAC/GTTC modification sites in EcoWI-seq. Data points indicated the threshold cutoff used. (TIF) [file pgen.1010389.s003.tif]

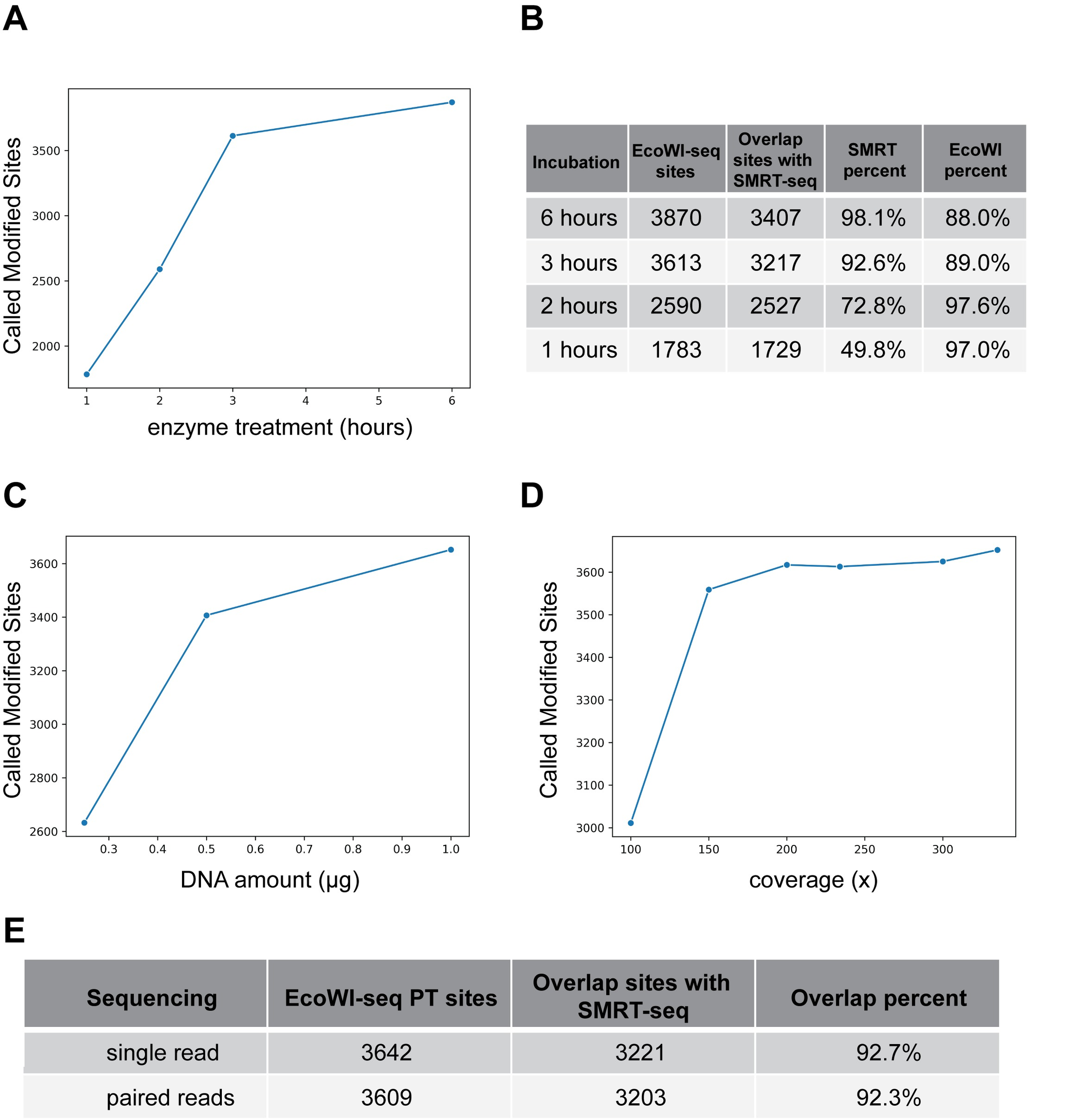

Supplement: S4 Fig — (A) Plot showing increased numbers of modified sites with increased incubation time. Data points represented 1, 2, 3 and 6 hours enzymatic treatments respectively. (B) Numbers of modified GAAC/GTTC sites and overlap percentage with different incubation conditions. Percent SMRT: percentage of SMRT-seq sites overlapping with EcoWI-seq sites; Percent EcoWI: Percentage of EcoWI sites overlapping with SMRT-seq sites. (C) Plot showing numbers of modified sites over different amounts of DNA input. Data points indicated DNA input of 250 ng, 500 ng and 1000 ng respectively. (D) Plot showing numbers of modified sites using different sequencing depth. Data points represented an average coverage of 100x, 150x, 200x, 234x, 300x and 335x respectively. (E) Numbers of modified GAAC/GTTC sites and overlap percentages by single end or paired-end sequencing. Reads were down-sampled to the same numbers for comparison in the two conditions. Overlap percent: Percentage of EcoWI sites overlapping with SMRT-seq sites. (TIF) [file pgen.1010389.s004.tif]

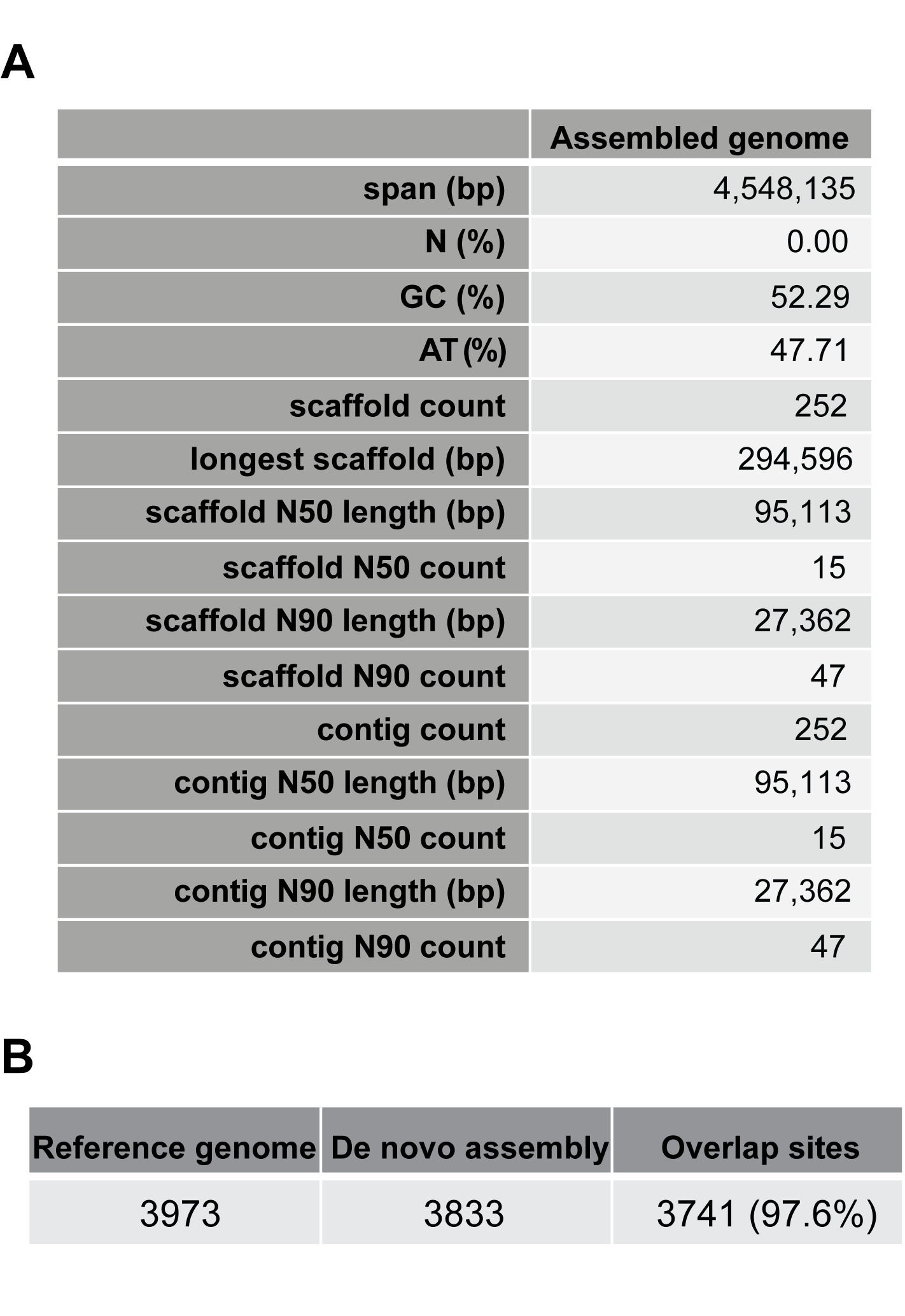

Supplement: S5 Fig — (A) Features of de novo assembly using SPAdes assembler. (B) Table listing numbers and overlapping of called modified sites between de novo assembly and using reference genome. (TIF) [file pgen.1010389.s005.tif]

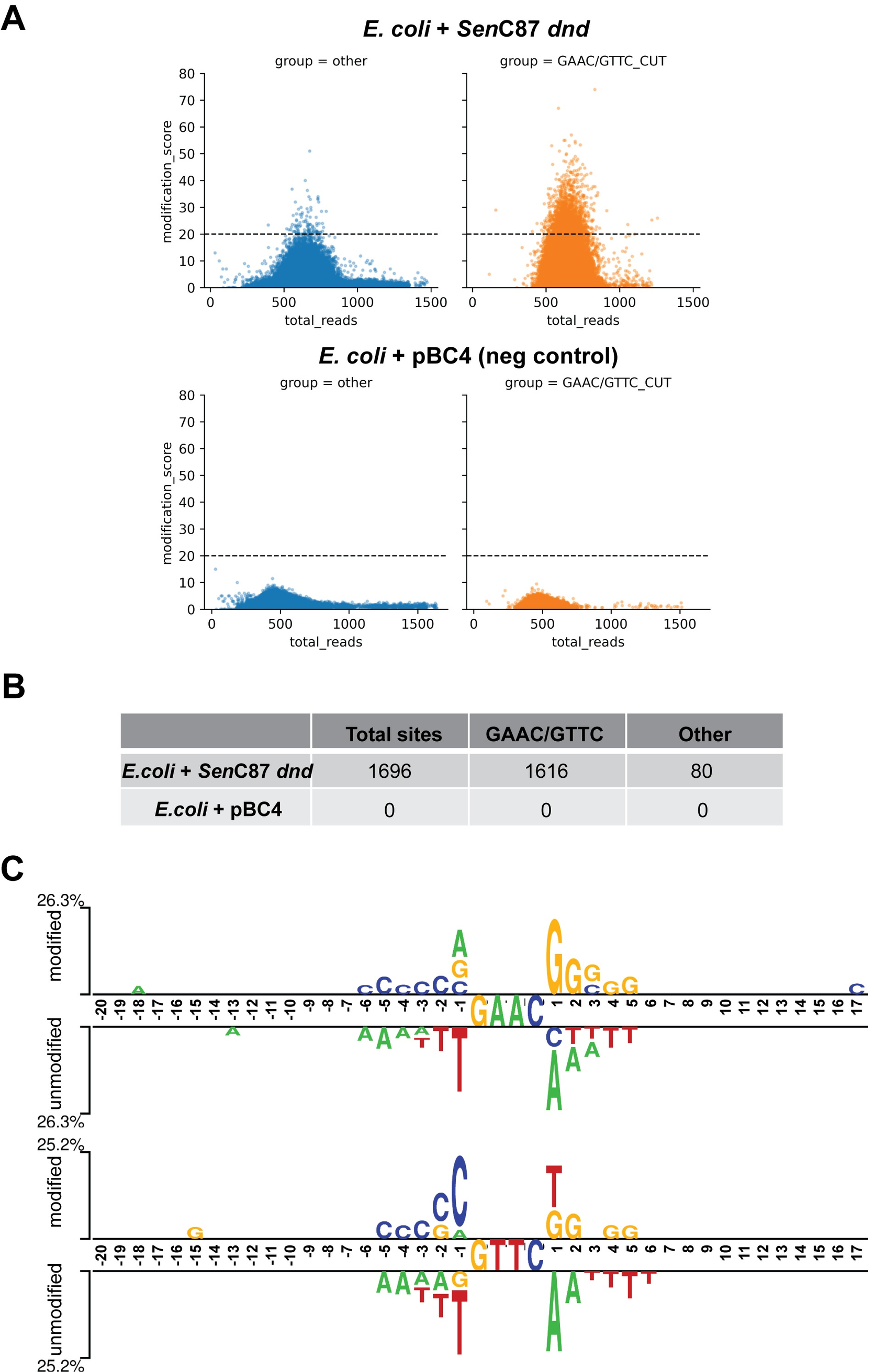

Supplement: S6 Fig — (A) Plots of modification scores in the genome of SenC87 dnd transformed and pBC4 transformed E. coli strains. Dashed line showed the modification score cutoff used to call PT modified sites. (B) Numbers of identified PT-modified sites by category. (C) Output of Two Sample Logo: Graphical representation showing statistically significant (p value < 0.01) differences in position-specific symbol compositions between modified and unmodified GAAC positions (top) and modified and unmodified GTTC positions (bottom) in the SenC87 genome. (TIF) [file pgen.1010389.s006.tif]
