# Supplementary material for: High-throughput sequencing of EcoWI restriction fragments maps the genome-wide landscape of phosphorothioate modification at base resolution
Source: PLoS Genet. 2022 Sep 19;18(9):e1010389. doi: 10.1371/journal.pgen.1010389 (PMC9521924; doi:10.1371/journal.pgen.1010389)
Supplement: Attachment — Submitted filename: answers_to_reviewers_Yang_et_al.docx [file pgen.1010389.s011.docx]

Reviewer #1: In this manuscript, the author developed EcoWI-seq based on a modification-dependent restriction endonuclease EcoWI to profile the phosphorothioation modifications in the genomic DNA. In the previous work, they characterized EcoWI as a PTDR to cleave the DNA strand at a fixed distance from its recognition sequence GpsAAC/GpsTTC. Here, they extended a straightforward application of EcoWI for genome-wide PT modifications identification at single base resolution combined with next generation sequencing. Similar to other sequencing methods based on various restriction endonuclease, EcoWI-seq is easy-operated and can be optimized to little starting material. The shortcoming of these kinds of method are obvious by the strand specificity of the restriction endonuclease. The comments and suggestions are listed below:

1. In the previous work, the author studied the performance of EcoWI in synthesized oligonucleotides and plasmids. It is suggested that these artificial DNAs with known modification ratios should be added to the experimental samples as spike-in. It will provide very clear evidence for the accuracy of EcoWI-seq.

This is a good point. We had initially designed an experiment with synthesized 200 bp oligonucleotides containing a PT modification in a GpsAAC/GpsTTC context as spike-in. Nonetheless, the results were not conclusive mainly due to oligonucleotide constraints. This is likely due to the fact that : [1] the binding of EcoWI to the modified oligonucleotides is hampered by the short length of the oligonucleotides. In addition, the digested fragments may be too short to enter the sequencing library; [2] organic synthesis of the PT modification leads to both right-handed (Rp) and left-handed (Sp) isomers. However, the enzyme has a preference for the naturally occurring isomer (Rp). Thus, in our hands, these oligonucleotides are not optimal for the validation of EcoWI-seq.

Instead, as the reviewer suggested, we used the pLacZZ-dnd+ plasmid as the spike-in in all of our sequencing experiments, including the *SenC87* and *E. coli* samples. In Figure 1 below, we show an example PT modified loci (GpsTTC) on the plasmid to demonstrate that EcoWI-seq distinctively generates pile-ups of reads only in the enzyme treated sample but not in the no enzyme control. For every experiment, the success of EcoWI-seq analysis on spike-in plasmid was first confirmed before any further analysis was performed. We revised the manuscript to specify this information:

“One µg genomic DNA was used for each reaction. The pLacZZ-dnd+ plasmid was spiked in as positive control. For transformed *E. coli* samples, Lambda gDNA spike-in were added to serve as negative control”

Figure 1. Genome browser (IGV) screenshot of a genomic region at a PT modified GTTC site (center) on pLacZZ-dnd+ plasmid. Reads from the EcoWI-seq (above) and control experiment (without enzyme, below) are mapping to the forward strand (pink) or reverse strand (blue).

2. There are mainly four types of consensus sequences for PT modification. EcoWI-seq can only detect one of them. Thus, what is the proportion of GpsAAC/GpsTTC in all sequences with PT modifications? It is better to tell the readers the percent of the detected sites among all PT modifications.

Bacteria with multiple dnd specificities have not yet been found suggesting that bacteria strain tend to have one single specificity. For bacteria with GAAC/GTTC specificity, EcoWI-seq will uncover all PT sites. We performed a literature survey and summarized 19 bacteria strains with verified PT specificities (see Table 1 below). From this, 7 (37%) have GAAC/GTTC specificity. Thus, if used on a mixed population of bacteria, EcoWI-seq is expected to uncover PT sites for about 37% of the bacteria with PT modifications. We have added this information together with the Table 1 below (also in the manuscript as Supplementary Table S4) in the discussion section of the manuscript:

“So far, four types of consensus sequences for PT modification have been identified in bacteria and archaea, namely, GpsAAC/GpsTTC, GpsGCC, GpsATC and CpsCA (hemi-modified on one strand only)[(Wang et al. 2011)](https://paperpile.com/c/Wv2KKs/Gpc5u). EcoWI-seq can only be used on bacteria with GAAC/GTTC consensus sequences for PT modification which accounts for ~37% of characterized PT containing bacteria strains (7 out of 19 strains, Supplementary Table S4) [(Wang et al. 2011; Cao et al. 2014; Wu et al. 2020)](https://paperpile.com/c/Wv2KKs/Gpc5u+hTVz+Q24JY).”

| **Organism** | **PT consensus motif** |
| --- | --- |
| *Pseudomonas fluorescens* PfO-1 | GGCC |
| *Vibrio splendidus* ZS139 | GGCC |
| *Enterovibrio calviensis* 1F-230 | GGCC |
| *Geobacter uraniireducens* Rf4 | GGCC |
| *Streptomyces lividans* 1326 | GGCC |
| *Hahella chejuensis* KCTC 2396 | GATC |
| *Halobellus limi* JCM 16811 | GATC |
| *Halapricum salinum* JCM 19729 | GATC |
| *Haloterrigena jeotgali* A29 | GATC |
| *Natronorubrum bangense* JCM 10635 | GATC |
| *Bermanella marisrubri* RED65 | GATC |
| *Shewanella pealeana* ATCC 700345 | GAAC/GTTC |
| *Escherichia coli* B7A | GAAC/GTTC |
| *Salmonella enterica cerovar Cerro* 87 | GAAC/GTTC |
| *Vibrio tasmaniensis* 1F-267 | GAAC/GTTC |
| *Vibrio cyclitrophicus* ZF65 | GAAC/GTTC |
| *Vibrio crassostreae* 9ZC77 | GAAC/GTTC |
| *Candidatus Pelagibacter ubique* HTCC1002 | GAAC/GTTC |
| *Vibrio cyclitrophicus* FF75 | CCA |

Table 1 (also as Supplementary Table S4 in the revised manuscript). Bacteria strains containing PT modification and their corresponding PT consensus motifs.

3. It is interesting that GpsTAC motif can be detected by EcoWI-seq besides the canonical GpsAAC/GpsTTC motif. The authors think that it may be attributed to the star activity of the EcoWI enzyme for either unmodified GTAC or PT modified GpsTAC motif. The biochemistry experiments should be performed to verify this possibility.

As the reviewer suggested, we synthesized 58 bp double-stranded oligonucleotides containing GpsTAC and performed EcoWI cleavage assay. The result demonstrates that EcoWI has partial activity on PT modified GpsTAC motif but not on unmodified GTAC motif. We therefore added this result as Supplementary Figure S2E and revised the manuscript :

In the Result section :

“It had been previously reported that some restriction enzymes (e.g. BamHI) could cleave some related sites by star activity [(George and Chirikjian 1982)](https://paperpile.com/c/Wv2KKs/PLdqi). To investigate if this is the case of EcoWI, we designed oligonucleotides containing modified GpsTAC motif or unmodified GTAC motif as control and performed EcoWI cleavage assay (Supplementary Figure S2E). The GpsTAC oligonucleotide is partially cut while the unmodified GTAC oligonucleotide is not digested at high enzyme concentration, demonstrating that EcoWI has activity on modified GpsTAC but not on unmodified GTAC motif. “

In the Discussion section:

“One limitation of the EcoWI-seq is that this method only recognizes PT modification in the GpsAAC/GpsTTC motif due to the specificity of the EcoWI enzyme. The activity of EcoWI on modified GpsTAC suggests either a slight relaxation of PT-dependent endonuclease activity or the intrinsic property of the enzyme. ”

In the Material and methods section:

“EcoWI digestion of PT-modified oligos (GpsTAC/GpsTAC)

58mer deoxyoligonucleotides (oligos) were purchased from IDT (Integrated DNA Technologies. USA). Below is shown the DNA sequences:

Top: 5’-CCTCGAGGTCGACGGTATCGCCCGATGpsTACCTAGTCGAATTCCTGCAGCCCGGGGGA-3’

Bottom: 5’-TCCCCCGGGCTGCAGGAATTCGACTAGGpsTACATCGGGCGATACCGTCGACCTCGAGG-3’

10 ng of the duplex DNA oligos (9 nM) containing PT-modified site (GpsTAC/GpsTAC) or unmodified site (GTAC/GTAC) were digested by 5.1 µg (76 µM) and 1.7 µg (26 µM) of EcoWI in NEB Buffer 2.1 for 2 h at 37 °C in a 30 µl reaction volume. The digest mix was treated with 4 ul of Proteinase K (3.2 U) at room temperature for 20 min. Half of the digested DNA was then analyzed by PAGE on a 10% TBE-PAG (Thermo-Fisher). DNA fragments were stained by SYBR Gold (Thermo-Fisher) and imaged on a Typhoon imager (GE Health). The same duplex oligos were also digested by EcoRI-HF (RI, 40 U, NEB) as positive digestion control for 2 h at 37 °C in NEB CutSmart buffer. 5-bp DNA ladder was purchased from Thermo-Fisher.”

Reviewer #2: Yang et al present a manuscript titled “High-throughput sequencing of EcoWI restriction fragments maps the genome-wide landscape of phosphorothioate modification at base resolution”. The manuscript described a new technique EcoWI-seq to detect phosphorothioate (PT) modification. The method relies on the specific restriction site of EcoWI enzyme that will cut GAAC/GTTC motifs of non-PT modified sites of genomic DNA. The manuscript is very well written and demonstrate a good experimental design to show the robustness of the technique. The authors used Salmonella enterica cerro 87 as the show case and showed a high consistency result with previously published work of the same species. Moreover, the author also demonstrated the PT detection by the EcoWI-seq in the E. coli expressing the dnd+ gene cluster. The authors also provided a bioinformatic pipeline to analyze the data generated from EcoWI-seq in GitHub that will be useful for the community and for reproducibility of their work.

Comment.

- Sonication of EcoWI digested DNA could produce a background noise due to a probability of the DNA breaking at the GAAC/GTTC with PT modified sites. Please discuss the impact of sonication.

Sonication has the property of breaking canonical DNA in a more or less randomized manner. The reviewer is right in wondering whether the sonication has a propensity to break the DNA preferentially at PT modified sites. This would lead to a pile-up of reads at GpsAAC/GpsTTC sites (between G and A or G and T) in addition to the enzyme cut sites which are a few bases away. However, this is not common in our datasets. In Figure 2 below, we show a genome viewer screenshot from a genomic region spanning a modified GpsAAC/GpsTTC site. The central line marks the position of the G in the GAAC motif and the enzyme cut site in the upstream is also marked. Neither the EcoWI treated sample (top panel) nor the no enzyme control sample (bottom panel) has the pile-up of reads at the potential breakpoint (between G and A). Conversely, a clear pile-up of forward and reverse reads can be observed at the expected enzyme cut site only in the treated sample. To generalize this observation, we also performed a genome-wide analysis of the GAAC/GTTC sites and calculated the modification scores at the potential breakpoints (Figure 3 below). While a small number of these sites have high modification scores (or pile-up of reads), it turns out that the majority of them were due to two adjacent GAAC/GTTC sites spaced at exactly the distance so that the enzyme cut site coincides with the other GAAC/GTTC site (those cases are marked in orange). For example, 44772 is a GAAC position, and it’s also a cut site of 44761 GTTC. Very few of the GAAC/GTTC sites showed pile-ups that are likely generated by sonication (marked in blue). Together, these results indicate that the sonication had no propensity to break the DNA at GpsAAC/GpsTTC sites and therefore the sonication step is unlikely to affect the performance of EcolWI-seq.

Figure 2 : Genome browser (IGV) screenshot of a genomic region (*SenC87*) at a PT modified GAAC site (center). Reads from the EcoWI-seq (above) and control experiment (without enzyme, below) are mapping to the forward strand (pink) or reverse strand (blue).

Figure 3 : Scatterplot showing the modification scores of all GAAC/GTTC positions in the *SenC87* genome. Each dot represents a GAAC/GTTC position and is colored by whether it coincides with the enzyme cut site of an adjacent GAAC/GTTC motif. Top: EcoWI treated sample; Bottom: no enzyme control.

- Figure 3b is conclude nothing to me. We cannot see any details of base per base comparison due to too much information. I recommend remove from the panel main figure.

As suggested by the reviewer, we moved Fig 3B to supplementary Figure S3B.

- The results from E. coli expressing the dnd+ gene cluster can be used to understand a mechanism of PT modification. I would like the authors explore more what’s happened in E coli compare to Salmonella and report in the manuscript such as i) sequence context around of PT modification sites, ii) gene that has PT modification site(s).

i) sequence context around of PT modification sites

We thank the reviewer for this comment to improve our analysis and manuscript. For the first suggestion, we have already analyzed the sequence variability (+/- 20 bp) around the PT modified sites using the differential sequence logo between modified and unmodified sites (see the manuscript Figure 4C). We reported the result in the manuscript, more specifically :

“We observed nucleotide preferences immediately upstream and downstream of the modified GAAC/GTTC motif with C and G respectively are over-represented in the modified motifs at -2 and +2 positions relative to the modified compared to unmodified GAAC/GTTC motifs. The -1 bp and +1 bp positions at modified GAAC/GTTC motifs have preference for M (A or C) and K (T or G) respectively (Figure 4C).”

To more directly address the reviewer’s question we also perform a regular motif logo analysis (see figure 4 below) : In agreement with the differential sequence logo analysis, we observed similar sequence preference at -2/+2 positions from the modified GAAC/GTTC motif (-2 bp prefers a C; +2 bp prefers a G).

Figure 4. Motif logo of modified GpsAAC/GpsTTC positions in SenC87 dnd transformed E. coli strains, as identified by EcoWI-seq.

ii) gene that has PT modification site(s).

For the latter suggestion, we conducted analysis to investigate the distribution of PT modifications in different genomic regions (See Figure 5 below). In accordance with the literature, we do not see major preferences for a certain type of genomic features [(Cao et al. 2014; Tong et al. 2018)](https://paperpile.com/c/Wv2KKs/hTVz+GJ0u). We also performed a GO (molecular function) analysis and found a small preference for PT modification in notably ATP hydrolysis activity, nucleotide binding activity and transporter activity related genes (See Figure 6 below). These figures will not be included in the manuscript but are attended only for reviewer’s evaluation since these results have been published in the literature.

Figure 5. The distribution of PT modifications in genomic regions. CDS: coding sequence; ncRNA: non-coding RNA; other: other regulatory regions

Figure 6. Gene ontology analysis of genes containing PT modifications in *SenC87 dnd* transformed E. coli strains

Reference:

[Cao, Bo, Chao Chen, Michael S. DeMott, Qiuxiang Cheng, Tyson A. Clark, Xiaolin Xiong, Xiaoqing Zheng, et al. 2014. “Genomic Mapping of Phosphorothioates Reveals Partial Modification of Short Consensus Sequences.” Nature Communications. https://doi.org/](http://paperpile.com/b/Wv2KKs/hTVz)[10.1038/ncomms4951](http://dx.doi.org/10.1038/ncomms4951)[.](http://paperpile.com/b/Wv2KKs/hTVz)

[George, J., and J. G. Chirikjian. 1982. “Sequence-Specific Endonuclease BamHI: Relaxation of Sequence Recognition.” Proceedings of the National Academy of Sciences of the United States of America 79 (8): 2432–36.](http://paperpile.com/b/Wv2KKs/PLdqi)

[Tong, Tong, Si Chen, Lianrong Wang, You Tang, Jae Yong Ryu, Susu Jiang, Xiaolin Wu, et al. 2018. “Occurrence, Evolution, and Functions of DNA Phosphorothioate Epigenetics in Bacteria.” Proceedings of the National Academy of Sciences of the United States of America 115 (13): E2988–96.](http://paperpile.com/b/Wv2KKs/GJ0u)

[Wang, Lianrong, Shi Chen, Kevin L. Vergin, Stephen J. Giovannoni, Simon W. Chan, Michael S. DeMott, Koli Taghizadeh, et al. 2011. “DNA Phosphorothioation Is Widespread and Quantized in Bacterial Genomes.” Proceedings of the National Academy of Sciences of the United States of America 108 (7): 2963–68.](http://paperpile.com/b/Wv2KKs/Gpc5u)

[Wu, Xiaolin, Bo Cao, Patricia Aquino, Tsu-Pei Chiu, Chao Chen, Susu Jiang, Zixin Deng, et al. 2020. “Epigenetic Competition Reveals Density-Dependent Regulation and Target Site Plasticity of Phosphorothioate Epigenetics in Bacteria.” Proceedings of the National Academy of Sciences of the United States of America 117 (25): 14322–30.](http://paperpile.com/b/Wv2KKs/Q24JY)
